# Prolonged sexual abstinence after childbirth: gendered norms and perceived family health risks. Focus group discussions in a Tanzanian suburb

**DOI:** 10.1186/1472-698X-13-4

**Published:** 2013-01-15

**Authors:** Columba K Mbekenga, Andrea B Pembe, Elisabeth Darj, Kyllike Christensson, Pia Olsson

**Affiliations:** 1 Department of Community Health Nursing, School of Nursing, Muhimbili University of Health and Allied Sciences, Dar es Salaam, Tanzania; 2 Department of Women’s and Children’s Health, Uppsala University, Uppsala, Sweden; 3 Department of Obstetrics and Gynaecology, School of Medicine, Muhimbili University of Health and Allied Sciences, Dar es Salaam, Tanzania; 4 Department of Women’s and Children’s Health, Karolinska Institute, Stockholm, Sweden

**Keywords:** Prolonged sexual abstinence, After childbirth, First-time parents, Gender relations, FGD, Tanzania

## Abstract

**Background:**

Prolonged sexual abstinence after childbirth is a socio-cultural practice with health implications, and is described in several African countries, including Tanzania. This study explored discourses on prolonged postpartum sexual abstinence in relation to family health after childbirth in low-income suburbs of Dar es Salaam, Tanzania.

**Methods:**

Data for the discourse analysis were collected through focus group discussions with first-time mothers and fathers and their support people in Ilala, Dar es Salaam, Tanzania.

**Results:**

In this setting, prolonged sexual abstinence intended at promoting child health was the dominant discourse in the period after childbirth. Sexual relations after childbirth involved the control of sexuality for ensuring family health and avoiding the social implications of non-adherence to sexual abstinence norms. Both abstinence and control were emphasised more with regard to women than to men. Although the traditional discourse on prolonged sexual abstinence for protecting child health was reproduced in Ilala, some modern aspects such as the use of condoms and other contraceptives prevailed in the discussion.

**Conclusion:**

Discourses on sexuality after childbirth are instrumental in reproducing gender-power inequalities, with women being subjected to more restrictions and control than men are. Thus, interventions that create openness in discussing sexual relations and health-related matters after childbirth and mitigate gendered norms suppressing women and perpetuating harmful behaviours are needed. The involvement of males in the interventions would benefit men, women, and children through improving the gender relations that promote family health.

## Background

Sexuality as the social construct of a biological drive is closely related to gender, another social construct. Men and women are socialised to culturally- specific and socially acceptable ideals of masculinity and femininity [1], and the period after childbirth is no exception. Sexuality has long been a subject of secrecy and taboo in Africa [2]. However, the presence of HIV has precipitated the move towards more openness on how sexuality is conceptualised [3,4], and gendered norms on sexuality and decision-making have been dismantled [5-8].

Prolonged sexual abstinence after childbirth is a socio-cultural practice with health implications and is described in several African countries. The practice of postpartum abstinence is closely linked to child spacing in Tanzania [9], Ghana [10], and the Ivory Coast [11,12], with additional connections to lactation and child health in Ghana [10], the Ivory Coast [11], and Malawi [13]. In Tanzania, the 2010 demographic and health survey [14] found the median length of postpartum abstinence to be 3.8 months, with 19% of participants in the survey still abstaining at 12–13 months postpartum. This is about half the median length of postpartum abstinence (6.5 months) estimated 20 years earlier [15] and indicates a marked reduction in postpartum abstinence over the years, although the reasons for abstinence are not presented.

Despite the decrease in the length of sexual abstinence postpartum, 50% of first-time mothers worry both over the timing of resuming sex, and how their partner will react to both them and the baby, six weeks after childbirth [16]. Qualitative interviews in the same area reveal that both women [17] and men [18] express concerns over the resumption of sex during the breastfeeding period, with a belief this may negatively affect the health of the baby. In Ghana [10], Ivory Coast [19], and Nigeria [20], there is an increased risk of the spread of HIV and other sexually transmitted infections (STIs) during the breastfeeding period, as men are reported to engage in extramarital sex while their women partners observe abstinence. Such risks and concerns over the possible health implications are also voiced by Tanzanian first-time mothers [17] and fathers [18]. However, different countries and communities, have differing and context-specific explanations for postpartum sexual abstinence and its social and health implications for the family. Thus, a deeper understanding is required about how the parents and community at large perceive sexual relations after childbirth in relation to health as a contribution to reducing the knowledge gap in the study setting.

Traditionally, support after childbirth in Tanzania is provided by family members [16]. However, young populations in urban areas are less likely to receive family support and more likely to experience divergent views from different ethnic groups they encounter, as they attempt to adapt to their new urban settings [21]. Thus, an exploration of this population would provide a deeper understanding of health and other socio-cultural challenges facing first-time parents after childbirth.

This study therefore explored discourses on prolonged sexual abstinence after childbirth in relation to family health in low-income suburbs of Dar es Salaam, Tanzania. The description provides a comprehensive picture that can be used as a resource for policy deliberations on how sexual education, postpartum care, contraceptive services, and HIV/STI prevention can be improved.

## Methods

Qualitative methods are suitable for exploring people’s understanding of social phenomena from their own perspective [22]. Data were collected through focus group discussions (FGD) [23] and analysed through discourse analysis inspired by Parker [24].

### Study setting

This study was undertaken in the low-income suburbs of Ilala, a municipality comprising 637,000 of the 2,487,300 inhabitants that make up the total population of Dar es Salaam [25]. The majority of inhabitants of Illala are internal migrants and represent the multiple cultures and ethnic groups that make up Tanzania [26]. In Ilala, unemployment is high and most young adults are self-employed in petty businesses that are often insufficient to meet basic needs [27]. Hence, Ilala shares the same characteristics as many low-income suburbs in cities of low-income countries: a young, sexually active, and fertile population, poverty, high congestion, poor sanitation, poor health, and insufficient transport system [21,26].

In Tanzania, health care after childbirth focuses on family planning services and the prevention of childhood illnesses. There is no formal health care follow-up for either the woman or her partner after uncomplicated childbirth, which implies that many health concerns during this period are unattended by health professionals. As many as 90% of women in Dar es Salaam deliver in health care facilities: nationally, only 51% of women deliver in health care facilities [14].

### Participants and recruitment

Government community leaders, including street leaders, were informed about the aim and procedures of the study and asked to help identify participants. The criteria for selection included being a first-time mother or father who cohabited with their partner and infant of 6 months of age or under, and women and men with experience of supporting first-time parents, such as grandmothers and grandfathers, in-laws, aunts, maternal and paternal parents, and neighbours. Purposive sampling was used [22] to capture variations in perceptions relating to experience and gender. A snowball approach [22] was later used to obtain subsequent participants. The recruitment resulted into eighty-two (82) participants from 29 different ethnic groups. Details on the compositions of the FGDs are found in Table 1.

**Table 1:** Participants and focus group discussions (FGD)

**Participants**	**Age range (years)**	**Average age (years)**	**Number of participants**	**FGD conducted**	**FGD analysed**
Mothers	18-30	21	24	4	3
Fathers	21-31	26	16	3	3
Female supporters	38-70	54	21	4	4
Male supporters	42-90	63	21	3	3
TOTAL			82	14	13

### Focus group discussions

Between August 2009 and January 2010, fourteen FGDs were conducted with 4 to 8 people participating in each FGD. To facilitate group interactions, the participants were allocated to groups based on gender and age [23]: the compositions of the focus groups are presented in Table 1.

A piloted hypothetical scenario [23] was constructed based on previous interview studies with mothers [17] and fathers [18], and initially read to the groups. The scenario highlighted family health and practices, and sexual and gender relations after childbirth. The first, female, author (CKM) moderated the FGDs with women, and the second, male author (ABP) moderated the FGDs with men. All FGDs were conducted in Kiswahili, which was fluently spoken by all participants. The discussions were audio recorded and field notes were taken.

The scenario presented at the beginning of each FGD

Neema is a young Chagga [ethnic origin] woman who is self-employed with a small hair salon in Buguruni. She is married to Peter and they were blessed with their first baby four months ago. Neema’s husband, the young father Peter, is Hehe [ethnic origin] and a self-employed shopkeeper. He is very excited about their baby and feels guilty for not spending enough time with his new baby and partner. However, he cannot manage, as his family depends on his daily earnings from his small shop.

As this was their first baby, they were not sure how to take care of the cord and how Neema’s hygiene and nutritional status should be maintained. Neema was also worried because she had vaginal discharge a few days after delivery. Neema’s mother-in-law applied hot water compressions, but Neema complained that it was very hot and uncomfortable.

Neema is breastfeeding exclusively, as advised by the midwives. However, the baby has been crying, especially at night. They do not sleep much and feel stressed. Neema and Peter suspect that the baby is not getting enough breast milk and they are wondering whether they should start her with light porridge.

Peter wants them to resume sex, but Neema maintains they should abstain until the child has stopped breastfeeding i.e. one to two years. They had a big argument, where Peter slapped her because she refused him sex. Neema worries Peter might not abstain and be unfaithful.

### Data analysis

The FGDs were transcribed in Kiswahili and translated to English before analysis. One FGD with mothers was not possible to analyse due to poor sound quality. The first author listened to all recordings and compared them with transcripts in both languages to ensure the quality of data transformation. The transcripts were analysed by discourse analysis [24]. *‘Discourse analysis deliberately systematizes different ways of talking so that we can understand them better’*[24] page 5. After reading the text for general understanding, a closer and detailed analysis identified all relevant statements referring to prolonged sexual abstinence after childbirth. These were later sorted into different categories as they were reviewed. The analysis included the determination of which people were being referred to, which people gained or lost from the utilisation of certain aspects in the discourse from the health and gender perspectives, and the connections between discourses.

### Ethical issues

Ethical approval for this study was granted by the Senate Research and Publications Committee of the Muhimbili University of Health and Allied Sciences, Dar es Salaam, Tanzania. Permission was granted by the office of Municipal Director, Ilala Municipality. The Regional Ethical Review Board of Uppsala University, Uppsala, Sweden, conducted a consultative review. Oral information on the aim and procedures of the study was provided in Kiswahili to all potential participants. Verbal consent was obtained, and participants were reassured of confidentiality. Participants were asked to reveal only what they felt comfortable sharing with others, as investigators could not guarantee confidentiality among participants. Participants were free to leave the discussions or decline to answer any question at any point, without being required to give reasons. No participant left, but some remained silent during parts of the discussions.

## Results

From the FGDs, three interconnected discourses on prolonged sexual abstinence after childbirth emerged (Table 2). In the quotes from the FGDs, ‘M’ stands for moderator and ‘P’ for participant, ‘SP Females’ for female support people, ‘SP Males’ for male support people, and the numbers indicate different participants in the FGD (e.g. P2) and distinguish the different groups (e.g. FGD5).

**Table 2:** Discourses on prolonged sexual abstinence after childbirth

1	Sexual abstinence to avoid ‘*kubemenda*’
2	Control of sexual desire and the subjective positioning of men and women
3	Traditional discourse being challenged by modernity

### Sexual abstinence to avoid ‘kubemenda’

#### Kubemenda

The dominant discourse on sexual abstinence after childbirth was about the importance of abstaining from sexual intercourse in order to protect the baby from getting *kubemenda. Kubemenda* was described as a phenomenon in which a child has poor growth and development, and ill health resulting from the parents’ non-adherence to sexual abstinence*.* A child affected by *kubemenda* could be recognized as it presented with different symptoms, the most prominent ones being frequent diarrhoea, malnutrition, abnormal thinness, poor growth, weak upper and lower limbs, generalised body weakness or paralysis. Less prominent symptoms included shiny cheeks, swollen limbs, and convulsions. The possibility that *kubemenda* did not exist as a childhood condition related to the parents’ non-adherence to sexual abstinence was not mentioned in any FGD. This implied a strong belief in the existence of this phenomenon.

P: As you know when a woman is taking care of the child, she is not supposed to have sex with her husband because the child’s health will deteriorate; sometimes the child might get convulsions […]. FGD 3 – Mothers

#### Constructions of ‘kubemenda’

There were several ways of explaining why *kubemenda* appears. It was often described as occurring when the mother had sexual intercourse during the breastfeeding period. Another reason was that the woman conceived during the breastfeeding period. In addition, *kubemenda* could occur because one of the couple had extramarital sex. Interestingly, some participants described men’s extramarital sex as a way of protecting the child from *kubemenda*.

Three constructs on the mechanisms causing *kubemenda* were revealed: heat or sweat produced during sexual intercourse; sperm entering and contaminating breast milk; and, touching the baby without washing the body properly after having sexual intercourse.

P: When the mother washes herself [after having sex] and then breastfeeds the child, there is no problem. However, when you ejaculate inside and leave out the sperm in the vagina it seems the semen passes, we do not know where. We are not on a doctor’s level of understanding. However, later on, when the child breastfeeds, the semen enters the breast milk [they all say “breasts of the mother”] eeh! […] FGD 2 –Fathers

#### Meanings attached to sexual abstinence

In this discourse, sexual abstinence was given several meanings, ranging from total abstinence from sex for both parents, to occasional or less frequent sex, to the woman abstaining while the man has sex with another woman. Maintaining prolonged sexual abstinence during the period after childbirth was delineated as problematic, and at times, unrealistic and against human nature.

P: So the issue of abstaining sex. He can say okay to it, but practicing it while in the battlefield, staying in the same room with his wife where they used to make love. It will be a little bit of a problem. FGD 1 - SP Males.

The child can continue to breastfeed and you can continue to have sex with your husband. However, not every day, you can arrange to have sex once a week so that you do not endanger [cause *kubemenda*] the child. FGD 8 – Mothers.

There were variations on the exact period the couple should abstain from sex to ensure child’s health, ranging from 40 days to two and half years. Generally, the child’s growth and development indicated when sex could be resumed, such as when the child had been weaned or was able to crawl and walk.

M: How long should it take before they can have sex normally?

P1: They have to wait until the child is able to walk and has been weaned.

M: `So, until the child is able to walk, does this mean a year?

P2: Yes! Until the child stops breastfeeding and is able to walk.

P4: Others advise until the child is 6 months, others two years, but mostly two years and a half. FGD 3 – Mothers

The child’s health (and whether it presented with *kubemenda* or not) was a proxy for recognising the couple’s non-adherence to the norm of sexual abstinence. A child with *kubemenda* was not socially acceptable, and the social consequences of *kubemenda* for the couple included gossiping about their misbehaviour, shame, and social stigma. Therefore, avoiding *kubemenda* was important for maintaining family integrity and meeting social expectations in relation to sexuality and family health after childbirth.

#### Strategies for preventing ‘kubemenda’

Several strategies for preventing *kubemenda* were outlined. These included the use of withdrawal and modern contraceptives, especially condoms that prevented sperm from reaching and poisoning breast milk, washing the body after sex to prevent dirt from sweat from contaminating the child, traditional herbs, and sleeping in separate beds or a temporary separation. In the temporary separation situation, the woman and the child would move to her parents or in-laws until the child had grown sufficiently to allow the parents to resume sex. Separation was also a way of avoiding social shame, as disputes about sexual resumption were easily overheard in the neighbourhoods. Masturbation surfaced as a male strategy for preventing *kubemenda*, although when this point was probed, some male participants appeared uncomfortable discussing it. If all other strategies failed, divorce was mentioned as an option.

P5: Yes! You may decide to leave your husband in order to rescue your child.

M: So, you stay with your parents?

P5: Yes! You go back home [to the parents] to take care of your child because you are having trouble with your husband every night and people [neighbours] keep on listening to you [all laugh]. FGD 4 - SP Females

### Control of sexual desire and the subjective positioning of men and women

#### Gendered expectations on abstinence

In the discourse on prolonged sexual abstinence after childbirth, there were gender differences in relation to the control of sexual desire, with men and women being positioned differently. Men were mainly described as weak, with little ability for controlling their sexual urge, and thus could not be expected to abstain from sex for a prolonged period. Extramarital sex was often portrayed as normal conduct for men.

P1: In reality, there is no man who can endure abstinence! There is none!

M: There is no man who can abstain from sex?

P1: There is no one who can wait until you are through with breastfeeding the child. No one!

P2: For one year and a half! None! FGD 3 – Mothers

P: But, at the moment, in Dar es Salaam, there are many women. So, one may decide not to have sex with his wife so that he will not endanger his child. So, he has an extramarital affair instead. He thinks that if he has an affair it gives him relief. FGD 5 – Fathers

The father’s extramarital affairs were depicted as acts of responsibility towards the baby, as this protected the baby from *kubemenda*.

The women were expected to adhere to the norms of abstinence more than the men were. The women were mainly positioned as being strong in controlling their sexual urge and were expected to be able to abstain from sex for longer periods.

P: […] women can abstain very well if they will not think of that [sex] and you just go back to normal, mmh. You may think that I am lying, but that is how I live since I was four months pregnant until now. So, if I was able to abstain for that long, why should I fail? Note that I have a four-month-old child! FGD 3 – Mothers

In contrast to men, if women decided to have extramarital affairs to fulfil their sexual needs, they were considered deviant or promiscuous.

P4: Those men and women who are unsettled [having more than one sexual partner] can get HIV/AIDS. But, there are other prostitute women. They have a husband at home but still have extramarital relationships while they know that there is this disease. FGD 4 - SP Females

The possibility of women’s sexual urge inducing them to seduce their partner into having sex, despite their intention to adhere to the norm of abstinence, was also expressed.

P: You see someone with a baby and wearing more than ten strings of waist beads [beads are believed to attract a man], for whom are you wearing those beads while you have a baby? […] What do those beads signify? That you love him! Don’t they? […]. Now, when the husband sees you will he not get erect after seeing those beads? FGD 7 - SP Females

The women’s main described worry was losing their partners to other women, and contracting HIV and other STIs. This was emphasised in the discussion about men being the main source of HIV in families if they engaged in extramarital sex during this period.

P: When he decides to go for extramarital affairs, he will love two things; that is the woman and AIDS. Apart from AIDS, there are other minor diseases. […] And when he comes back to his wife, he can be carrying anything to his house. FGD 6 - SP Females

#### Women’s conflicting roles and men’s authority

Although men were described as weak in controlling their sexual desire, they were also depicted as controlling over women, and could force them to have sex against their will, even when the women wanted to uphold the sexual abstinence norm. As a result, women were sometimes described as ‘giving in’ to men’s sexual advances in order to protect their marriage, avoid divorce, or family violence, such as battering and rape.

Furthermore, women were depicted as constrained in this subjective position, as they struggled to fulfil social expectations both as mothers and as wives. Some men were said to punish women who denied them sex by not providing economic support, which would force the women into having sex against their will.

P3: When you go to bed, you might be arguing and there will be no peace. You might ask for money and he tells you ‘I don’t have’ while you see it [money] and he might not give you.

Several Ps: He will not give you! FGD 4- SP Females

Throughout the discourse, the mother was accorded responsibility for ensuring the growth and safety of the baby, whereas, the father was depicted as the financial provider for the family. Strong women and good mothers were portrayed as being firm and abiding by the sexual abstinence norm to protect the health of the baby, maintain family integrity, and avoid shame. Consequently, women who decided to resume sex prematurely were considered irresponsible mothers. In this subjective position, the woman was blamed if the child became affected by *kubemenda*.

P: The strict person there, who nurtures, is the mother […]. The father does not raise the child, man! How can he? The father does it [raise the child] from the outside [by having extramarital affairs], if he wants his child to be with him [survive]. FGD 9 - SP Males

Women often described pretending to be unaware of, or disregarding, their partners having other sexual partners as a strategy for achieving the normative construct of a good and submissive mother and wife.

P: But if you see a man is having an affair you just leave [do not trouble] him alone and take care of the baby. The most important thing is that you are getting money for spending at home and you are eating. You should not trouble him. You should concentrate on your business and you take good care of the baby […]. FGD 6 - SP Females

### Traditional discourse being challenged by modernity

Although traditional discourse on prolonged sexual abstinence for protecting the child’s health was reiterated in the FGDs in Ilala suburbs, modern aspects were also described.

#### A part of traditional discourse

Sexual abstinence after childbirth was described as part of an old tradition to which both men and women should adhere in order to prevent *kubemenda* and the associated social shame.

P: In those times when we were breastfeeding, you would not think of that [sex] and we didn’t have time for that. We made sure the child is able to walk first and then we started having sexual intercourse. FGD 4 - SP Females

Men’s difficulty in abstaining from sex for longer periods was described as true. Even in the olden days, extramarital affairs were part of the traditional discourse.

P: I have given birth to ten children […]. But, I could not stay for two years taking care of the baby without being together [having sex] with my husband. He would run away from me, he would have another woman. FGD 7- SP Females

The discussions revealed the discourse on prolonged sexual abstinence after childbirth appeared to originate from traditional societies where men could have several wives. However, polygamy in the traditional form was not often described as an alternative in Ilala.

P: People at that time were obedient. Even us, we were obedient but our youth today are not obedient. Therefore, there is no way out; you have to advise him ‘Eeh my son, you see, things are difficult! If I could, I would marry you to a second wife. You are a man; I would get you a second wife. I know a woman can abstain but a man cannot. So, what I am saying is that you must comply with doctor’s advice [on condom use]. Nowadays, there are condoms, you can have sex with your wife and the child will not be affected. […] So I advise you that go and take condoms have sex with your wife as you were doing before’. FGD 1 - SP Males

The participants highlighted how traditional discourses have been transferred from one generation to another through traditional rituals. Just before childbirth, elders shared their experiences and knowledge on sexuality and sexual abstinence after childbirth to the expectant mothers and fathers. This reproduction of old traditions was described as a continuous process, where young parents were given advice and guidance deemed necessary at any time after childbirth. Similar support is still being offered to parents in Ilala.

#### Modern discourse enabling people to sidestep the restrictions

The practice of prolonged sexual abstinence after childbirth was repeatedly expressed as outdated. Both young and old participants described young couples as violating the sexual abstinence norms, thus, putting the infants at risk for *kubemenda.*

P1: No, we do not wait, to say the truth.

(Laughter)

M: Eeeh, so what do you do?

P1: You discuss with your husband and do what you know and then you keep quiet. That’s all. When you go to your parents, they might ask *‘what has happened to the child’*, but you know the secret and you keep it in your hearts (laughs). FGD 8 - Mothers

Traditional aspects such as abstinence and *kubemenda* were present simultaneously with modern aspects such as contraceptives, HIV, and the use of health care services. Young couples were depicted as different from those of previous generations, as they had better knowledge of sexuality issues and of how to protect themselves against pregnancy, HIV, and other STIs. Access to condoms and other modern contraceptives was considered advantageous for young couples, and as acceptable strategies for avoiding *kubemenda*.

#### Weak position of the public health care services

The public health services were not discussed much in the FGDs. However, health care providers could be consulted for treating a child affected by *kubemenda* or for providing counselling on contraceptives, HIV, and other STIs. Despite representing the medical discourse, health care workers were sometimes described as conveyors of the traditions of prolonged sexual abstinence and *kubemenda*.

P: Maybe if you go there [at health facility], the nurses will tell you that now that you are taking care of the babies, do not sleep with your husband every day. There is hot water, boil some water, you go and sponge yourself two or three times, after that, you will not have the desire for Athuman [a fictive man’s name]… They say this several times. FGD 6 - SP Females

Furthermore, modern couples were said to consult elders for advice in resolving marital conflicts, discussing the timing of resuming sex, treating an affected child, and facilitating temporary separation of the couple to ensure prolonged sexual abstinence.

## Discussion

The present study provided a detailed analysis of the discourses on prolonged sexual abstinence after childbirth in a low-income Tanzanian suburb. The dominant and socially desirable discourse in this study setting delineated sexual abstinence as a means of protecting the infant from a perceived illness called *kubemenda*. As a dominant discourse [24], this limited what people could say. Stereotypic gender relations, with male dominance and female submission, were normative and depicted as essential for controlling sexuality to ensure family health and avoid the social implications of non-adherence to sexual abstinence. The medical discourse with aspects such as contraceptives, HIV, and the use of health care services, was less prominent. Other co-existing and conflicting discourses delineated abstinence as being against natural human sexuality, and resulting in practices that could endanger family relationships and health. This is especially problematic in the era of HIV/AIDS. Nevertheless, modern discourses were interspersed among traditional ones.

The promotion of child health through birth spacing is the main reason for observing abstinence in Tanzania [9,28], Ghana [10], Ivory Coast [11,19], and Nigeria [20]. In the present study however, abstinence was observed mainly to avoid *kubemenda*. The viewpoint that condoms prevent pregnancy but could not always protect the child from *kubemenda* supports this argument. However, as reported from Malawi [13], the participants in the present study often referred to modern couples as having the possibility to use condoms and other modern contraceptives to protect the child and/or avoid further pregnancy. The variety of explanations of how *kubemenda* occurred and was prevented could possibly be explained by the multitude of ethnic and cultural backgrounds among the population in Ilala and the FGDs. However, in all the FGDs, there appeared to be genuine worry about resuming sex after childbirth, although opinions on the duration of abstinence differed. The low usage of modern contraceptives among married women, 34% in Tanzania and 30% of women in the reproductive age in Dar es Salaam [14], is problematic. The reluctance to use contraceptives could possibly reflect parents’ fear of sexual resumption that might result in a new pregnancy sooner than desired, in addition to having little awareness of, and poor access to, contraceptive methods.

In contrast, in other instances, condoms were also described as an acceptable strategy for avoiding *kubemenda* during the postpartum period. This is because condoms were believed to prevent sperm from possible poisoning breast milk. The higher awareness and acceptability of condoms compared to other modern contraceptives seen in the present study results indicate the importance of raising awareness of and improving access to a variety of contraceptive methods to enable informed choice among men and women.

There are no medical reasons for refraining from vaginal intercourse after childbirth once the woman’s discharge has ceased and any wounds have healed [29], which generally happens 4–6 weeks after delivery. Establishing this knowledge in communities could facilitate women’s and men’s informed choices on the timing of the resumption of sex postpartum. Health workers have a responsibility for providing this knowledge, particularly to first-time parents, and information and the provision of modern contraceptives have the potential to diminish tensions related to sexuality after childbirth.

Prolonged sexual abstinence of up to two and a half years was described as being the predominant behaviour in both the olden days and at present, suggesting a reiteration of the discourse across generations, and illustrating the role of discourse in the production and reproduction of social practices in societies [30]. However, these results should be interpreted with caution, as what people say in a group discussion does not necessarily accord with how they behave in real life. In a group discussion, the expression of views is likely to adhere to socially acceptable norms in a particular social context, and this is supported by findings from the Tanzania health survey [14], where the median length of postpartum abstinence was only 3.8 months.

An imbalance in the power relations between men and women in sexual relationships was highlighted, and this corroborated other work on dominant male sexuality [1,7,31]. Men were accorded more power and fewer restrictions on sexual matters than were women, who were burdened with more responsibilities and blamed if expectations were not met. The differences in the way men and women were positioned in relation to sexuality illustrated ‘*power determines whose pleasure is given priority and when, how, and with whom sex takes place*’ [1], page 2. As men were presumed ‘weak’ and having an uncontrollable sexual desire, they were privileged to continued sex life, whereas, women were restrained from the possibility of enjoying and meeting their sexual needs. This reflected the traditional feminine and masculine stereotypes prevalent both in Tanzania [8] and globally [32]. These concepts encourage, perpetuate, and normalise infidelity in men, and reproduce gender inequalities with negative implications for family health, such as contraction of STIs and family breakups. Nevertheless, these stereotypic ideas require cautious consideration, as other, less prominent, patterns were highlighted. Quantitative studies are needed to explore the distribution of these perceptions and practices in the broader study population. Furthermore, females participated in upholding male dominance discourses, a factor that has been previously described [33], indicating how deeply these ideas are embedded in the participants’ culture and, as such, are regarded as normative.

Whereas men were positioned as ‘weak’ in abstaining from sex, women were expected to curb the problem by providing sex to avoid risking HIV/STIs, and to protect their marriage, even if this happens against their will. Women in this position lacked the right to decide when to have sex (‘*lack of bedroom power*’*)*[6] page 167. Female submission to male sexual dominance is a social expectation [31]. However, in Ilala, this problem was compounded by women’s economic dependence, as they were expected to disregard their partners’ infidelity so as not to be deprived of basic needs such as food. Thus, achieving equal gender-power relations is unrealistic if women are not economically empowered. Obtaining positive relationships among couples and practices that promote family health requires economically independent women and general sexuality education covering gender dimensions, in which maternal and child health services are important stakeholders.

Different moral standards related to sexual activity after childbirth were used to determine who were good mothers and women and good fathers and men, a fact that supported previous findings [6,31]. For instance, women having extramarital sex were labelled as deviant and irresponsible, whereas, for men, the same behaviour was socially tolerable and even expected. Similar differences in moral standards in this area are described by mothers [17] and fathers [18]: Haram [34] suggests ‘deviant’ women might be tired of repression, and find ways of expressing and meeting their sexual urge by adopting positions to take control of their lives. This could be an alternative interpretation of the findings in the present study.

Sexuality as a socially constructed phenomenon can be controlled and channelled in directions where it does least harm [33]. The tensions in the discourse in the present study indicated a possible opening for introducing interventions aimed at promoting family health after childbirth. Interventions in the form of dialogue between couples, community members in general, and health care providers would create a forum for discussing different discourses, the tensions created, and their relation to various practices that are important for family health after childbirth. Further, the dialogue would act as a forum for the provision of health information and discussion on gendered norms relating to sexuality during the childbearing period. Increasing the awareness of gendered feminine and masculine notions that can compromise the health of the families is important for enabling couples to make informed choices in relation to sexuality, and for creating opportunities for health workers to reflect on their own perspectives and uncertainties as part of both the traditional and medical discourses.

The discourses indicated a strong link between perceptions on sexual behaviour and ideas about the qualities that make a good parent. This could be an asset and a motivating factor for change towards promoting healthier lifestyles during the childbearing period. Change could be promoted by creating awareness of how previous discourses that might have supported health in other times and settings could now lead to ill health, due to changed circumstances, such as the abandonment of organised polygamy and the high prevalence of HIV; and of new possibilities, such as condoms, oral contraceptives and increased knowledge about sexual relations. One of the challenges would be discussing and questioning the perceived direct link between sexual activity and ill health in infants, in the face of the strong popular belief in *kubemenda*. The existence of discourses contradicting the dominant discourse of abstinence could be used to motivate discussions and interventions for influencing a positive change towards healthy behaviour.

To understand both sexual relations after childbirth and the tension resulting from conflicting discourses in connection to *kubemenda* and sexuality, participants relied on traditional discourses, and only partly on medical discourses. However, this depended on specific contexts and individual situations and preferences. In accordance with previous studies on mothers [17] and fathers [18], if the worries outlined in the discourses were not addressed, they could have negative health implications for both the baby and the parents. The increased risk of contracting HIV/STIs in the case of multiple sex partners was considered a threat, as was disagreements between the couple over sexual abstinence. Furthermore, common childhood illnesses might be confused with *kubemenda,* implying the child might be denied health care and taken to traditional healers instead. In the worst scenario, parents might decide to stop breastfeeding prematurely to be able to have sex, contrary to professional advice. However, participants in the present study did not describe this as an option. Premature mixed feeding or weaning increases the risk of diarrhoea, infections, malnutrition, and slow development in infants [35], which could be interpreted as *kubemenda* from a layman’s perspective.

Although emotional health problems were not explored or mentioned in the FGDs, these problems, especially depression, are internationally described as frequent among women and men, in general, and during the postpartum period in particular [36-38]. From the results of the present study, the worries, relational problems, violence, and ill health indicate a possibility of the development of emotional problems. There are few studies on emotional health after childbirth in Tanzania [39], and further studies are thus needed to explore the frequency and character of depression among parents during the childbearing years.

Currently in Tanzania, the health care system pays little attention to maternal or family health after childbirth, including sexual matters [40]. In the present study, health workers were said to be active in reinforcing traditional discourses, which implies a lack of expertise on sexual matters after childbirth. Health care providers should inform, and discuss sex resumption and related problems after childbirth, with the parents. Midwives and other health care providers are in a better position to intervene if provided the opportunity, in the form of guidelines, content and skills, for sexual education after childbirth. A crucial contribution would be putting the Tanzanian national policy [40] for postpartum health care visits into practice.

Descriptions of the participants’ characteristics, setting, along with some illustrative quotes, were provided to help in the assessment of the transferability of the results from the present study to other contexts. One limitation of this study was the design, which did not allow ethnic differences to be discerned, although 29 ethnics groups, out of about 130 existing in Tanzania, were represented, and the FGD groups comprised a mixture of participants with different ethnic backgrounds. Furthermore, the study did not include first-time parents with higher educational levels and higher socioeconomic status. Their inclusion could have generated different perspectives.

## Conclusion

Prolonged sexual abstinence after childbirth for protecting the infant’s health and avoiding *kubemenda* was the dominant and socially desirable discourse in this study setting. The medical discourse with aspects such as contraceptives, HIV, and the use of health care services was less prominent. Prolonged sexual abstinence after childbirth was managed through controlling sexuality to ensure both family health and the avoidance of social implications resulting from non-adherence to sexual abstinence. Discourses on sexuality after childbirth are vehicles in the reproduction of gender-power inequalities, where women are subjected to more restrictions and control than men are. Interventions that create both openness in discussing sexual relations and health-related matters after childbirth and mitigate gendered norms suppressing women and perpetuating harmful behaviours are needed. The involvement of males in the interventions would benefit men, women, and children through improving gender relations that promote family health.

## Competing interests

The authors declare no conflict of interest with any person or institution.

## Authors’ contributions

CKM was involved in the conceptualisation and design of the study, fieldwork coordination, data collection, data analysis and interpretation, and manuscript drafting; ABP was involved in the conceptualisation and design of the study, data collection, data interpretation, and critically revising the manuscript. ED and KC were involved in the conceptualisation and design of the study and critically revising the manuscript. PO was involved in the conceptualisation and design of the study, data analysis and interpretation, and the critical revision of the manuscript. All authors have read and approved the final manuscript.

## Pre-publication history

The pre-publication history for this paper can be accessed here:

http://www.biomedcentral.com/1472-698X/13/4/prepub
